# Effect of Tofacitinib on Hemostasis in Patients with Ulcerative Colitis: A Comparative Ex Vivo Study

**DOI:** 10.3390/ph18040557

**Published:** 2025-04-10

**Authors:** Cristina Sánchez-Sánchez, Fabio Suarez-Trujillo, Cristina Ramírez, Irene Soleto, Jorge Mercado, Macarena Orejudo, Paula J. Martínez, Celia Rubio Collado, Mar Orts, María Jesús Rubio Franco, Antonio Planas, Natalia Acedo, Nora Butta, María Chaparro, Javier P. Gisbert, Montse Baldán-Martín

**Affiliations:** 1 Hospital Universitario de La Princesa, Instituto de Investigación Sanitaria Princesa (IIS-Princesa), Universidad Autónoma de Madrid (UAM), Centro de Investigación Biomédica en Red de Enfermedades Hepáticas y Digestivas (CIBEREHD), 28006 Madrid, Spain; cristissanchez20@gmail.com (C.S.-S.); fabio.suarez.trujillo@gmail.com (F.S.-T.); ramicristina460@gmail.com (C.R.); irenesoletof@gmail.com (I.S.); jorge.mercado.hlp@gmail.com (J.M.); macaorejudo@gmail.com (M.O.); paula_jmg@hotmail.com (P.J.M.); javier.p.gisbert@gmail.com (J.P.G.); 2 Servicio de Hematología, Hospital Universitario de La Princesa, 28006 Madrid, Spain; crubioc@salud.madrid.org (C.R.C.); mjrubiofranco@gmail.com (M.J.R.F.); nataliaacedo@hotmail.com (N.A.); 3 Servicio de Anestesiología y Reanimación, Hospital Universitario de La Princesa, 28006 Madrid, Spain; mar.orts@gmail.com (M.O.); antonioplanas@hotmail.com (A.P.); 4 Grupo de Coagulopatías y Trastornos de la Hemostasia, Hospital Universitario de La Paz-IdiPAZ, 28046 Madrid, Spain; nbutta@hotmail.com

**Keywords:** tofacitinib, ulcerative colitis, platelet aggregation, platelet activation, thromboelastometry

## Abstract

**Background:** Tofacitinib is effective for refractory ulcerative colitis (UC), a chronic inflammatory disease of the colonic mucosa. However, its use has been associated with an increased risk of thromboembolic events, prompting regulatory restrictions. Understanding the pathophysiological mechanisms contributing to these potential risks is critical for patient safety. We aim to evaluate and compare ex vivo the effects of tofacitinib and anti-TNF on coagulation parameters and platelet function. **Methods:** Whole blood and platelet-rich plasma from 10 active UC (aUC) and 10 quiescent UC (qUC) patients and 10 healthy controls (HC) were spiked ex vivo with tofacitinib, anti-TNF (as comparator), or a sterile solution. Coagulation kinetics were measured by rotational thromboelastometry (ROTEM), platelet aggregation by aggregometry, and platelet activation by flow cytometry. The study was conducted at Hospital Universitario de La Princesa. **Results:** Flow cytometry showed increased expression of activation markers CD62P and CD63 and higher PAC-1 binding in platelets from both aUC and qUC patients incubated with either tofacitinib or anti-TNF versus no drug. No differences were found between the drugs. CD63 expression also increased in HC after drug exposure, with no differences between anti-TNF or tofacitinib. Platelet aggregation and coagulation parameters did not differ between tofacitinib, anti-TNF, and no drug in aUC, qUC, and HC. **Conclusions:** Tofacitinib does not alter platelet function or coagulation in UC patients under ex vivo conditions compared to anti-TNF. The increased thromboembolic risk observed in some populations treated with tofacitinib cannot be attributed to these factors in UC patients.

## 1. Introduction

Ulcerative colitis (UC) is a chronic inflammatory disease of the colonic mucosa that typically starts in the rectum and extends proximally [1,2]. Clinical manifestations include bloody diarrhea, abdominal pain, and extra-intestinal manifestations [3]. UC’s etiology is unknown but involves genetic, environmental, and immune factors. While first-line treatments include salicylates, immunosuppressive drugs, and corticosteroids, moderate-to-severe cases often require biologic therapies [4]. However, approximately 50% of patients do not achieve clinical remission with these agents [5].

Tofacitinib is an oral small-molecule Janus kinase (JAK) inhibitor that has become a therapeutic option for refractory UC cases [6]. Initially approved for rheumatoid arthritis and psoriatic arthritis, it has demonstrated effectiveness in both inducing and maintaining remission in UC patients. However, the safety profile of tofacitinib raises significant concerns. Its use in patients with rheumatoid arthritis has been associated with an increased risk of thromboembolic events, including pulmonary embolism and deep vein thrombosis [7]. In particular, data from ORAL SURVEILLANCE in patients diagnosed with rheumatoid arthritis aged over 50 years with cardiovascular risk factors indicated a higher incidence of pulmonary embolism and venous thrombosis among patients treated with tofacitinib compared those treated with anti-tumor necrosis factor (TNF) agents [8]. More recent analyses have further refined the risk assessment, identifying two distinct subpopulations of tofacitinib with different relative risks when compared to TNF inhibitors [9]. Furthermore, studies from a large randomized cardiovascular safety trial and an extended analysis of phase 3b/4 safety data demonstrated a higher incidence of major adverse cardiovascular events and cancer in patients with rheumatoid arthritis receiving tofacitinib compared to anti-TNF [10,11].

Due to the increased risk of severe thromboembolic events, the European Medicines Agency (EMA) and the United States Food and Drug Administration (US FDA) have issued warnings advising against the long-term use of tofacitinib for maintenance in UC patients with one or more risk factors for venous thromboembolism [12,13]. In cases where no appropriate alternative treatments are available, the risk and benefits of tofacitinib should be carefully evaluated before its long-term use is considered. Additionally, other JAK-signal transducer and activator of transcription (STAT) pathway inhibitors such as baricitinib have also been associated with an increased risk for thromboembolic events, suggesting a potential class effect of JAK inhibitors [14].

JAK inhibitors target the JAK and STAT pathway, which is crucial for signal transduction and transcriptional activation. This pathway plays a key role in various cytokines and growth factors, and is essential for hematopoiesis, inflammation, and immune functions [15]. The JAK-STAT pathway mediates over 50 signaling molecules, including cytokines, hormones, and growth factors [16]. Given the broad impact of JAK inhibitors on this pathway, their use may disrupt the balance between prothrombotic and antithrombotic effects, potentially leading to thrombotic complications and cardiovascular events [17]. Some studies suggest that JAK inhibition may reduce endothelial prothrombotic activation and leukocyte–endothelial proadhesive interactions [18]. However, other research highlights that JAK inhibitors may increase cytokine production associated with immunothrombosis and delay clot lysis, particularly in active rheumatoid arthritis [19]. Further studies have proposed that the thromboembolic events observed with JAK inhibitors may be due to their low selectivity and pan-JAK blockade [20,21].

Thromboembolic events, associated with high morbidity and mortality, involve a complex interplay between pro-coagulant, anticoagulant, and fibrinolytic factors [22]. To elucidate the underlying mechanisms contributing to the increased thromboembolic risk in patients receiving JAK inhibitors, and to identify potential preventive measures, this study aims to clarify the effect of tofacitinib (in comparison with anti-TNF agents) on platelet function and coagulation parameters in UC patients.

## 2. Results

### 2.1. Baseline Clinical Characteristics

A total of 30 individuals (10 with active UC (aUC), 10 with quiescent UC (qUC), and 10 healthy controls (HC)) were included in the study. No significant differences were observed in baseline characteristics of the study population such as age, sex, smoking status, and body mass index between the different study groups (Table 1).

### 2.2. Effects of Tofacitinib and Anti-TNF on Platelet Activation

**Figure 1:**
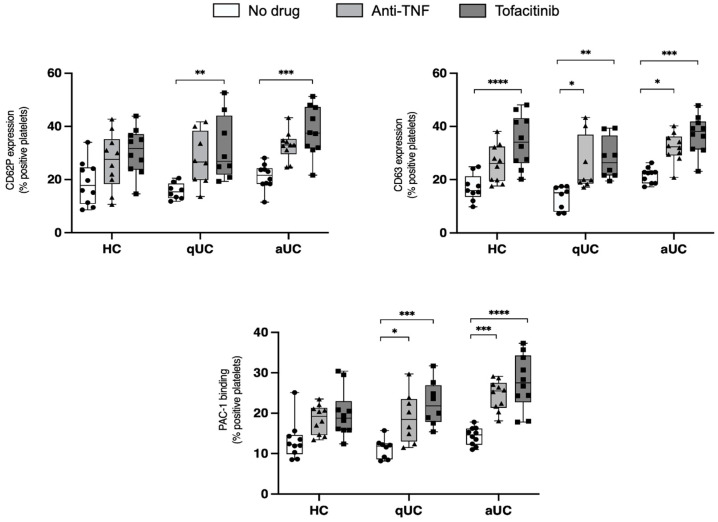
Expression of platelet activation markers in platelet-rich plasma samples under three different experimental conditions (tofacitinib, anti-TNF, or no drug) at baseline. Differences were analyzed with two-way ANOVA test. * *p*-value < 0.05, ** *p*-value < 0.01, *** *p*-value < 0.001, **** *p*-value < 0.0001. HC: healthy control; aUC: active ulcerative colitis; qUC: quiescent ulcerative colitis.

Platelets have an essential role in clot formation, playing a crucial part in both hemostasis and thrombosis. In the current study, the effect of tofacitinib and anti-TNF drugs on platelet activation in patients with aUC, qUC, and HC was evaluated. As observed in Figure 1, the results demonstrate that tofacitinib enhanced exposure of CD63 and CD62P and binding of PAC-1 in quiescent platelets from qUC and aUC, indicating an activating effect on platelets. CD63 is a lysosomal marker associated with granule release, CD62P (P-selectin) is a key molecule involved in platelet aggregation and interaction with leukocytes, and PAC-1 binding reflects the conformational activation of the fibrinogen receptor (GPIIb/IIIa), which is crucial for platelet aggregation. However, this effect does not appear to be specific to tofacitinib, as anti-TNF also produced the same effect on CD63 exposure and PAC-1 binding. Notably, while there is actually a general trend toward increased platelet activation in qUC and HC following exposure to tofacitinib and anti-TNF, the only statistically significant effect observed in these groups is the increase in CD63 expression.

**Figure 2:**
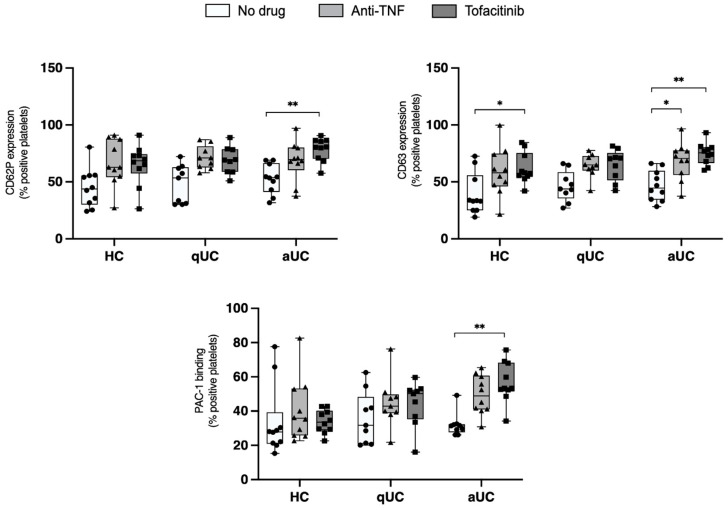
Expression of platelet activation markers in platelet-rich plasma samples under three different experimental conditions (tofacitinib, anti-TNF, or no drug) after stimulation with thrombin receptor activating peptide. Differences were analyzed with a two-way ANOVA test. * *p*-value < 0.05, ** *p*-value < 0.01. HC: healthy control; aUC: active ulcerative colitis; qUC: quiescent ulcerative colitis.

To further explore the functional responsiveness of platelets to strong agonists, their activation following stimulation with thrombin receptor activating peptide (TRAP) was assessed, as depicted in Figure 2. Consistent with the findings in Figure 1, we observed that both tofacitinib and anti-TNF drugs significantly increased CD63 expression on platelets from patients with aUC after stimulation with TRAP.

### 2.3. Effect of Tofacitinib and Anti-TNF on Platelet Aggregation

**Figure 3:**
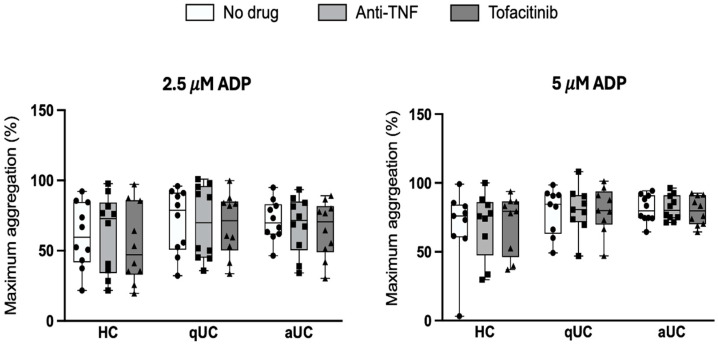
Effect of tofacitinib and anti-TNF on ex vivo platelet aggregation. Box plot shows maximum adenosine diphosphate-induced aggregation of platelet-rich plasma from participants of the study incubated 30 min at 37 °C with anti-TNF, tofacitinib, or no drug. Platelet-poor-enriched plasma was used as a blank. HC: healthy control; qUC: quiescent ulcerative colitis; aUC: active ulcerative colitis.

Maximum platelet aggregation induced by adenosine diphosphate (ADP) (2.5 µM and 5.0 µM) was assessed in three groups (HC, qUC, and aUC) under three experimental conditions (incubation with tofacitinib, with anti-TNF, and without drug). No significant differences in platelet aggregation were observed between the different experimental conditions in any of the groups (Figure 3).

### 2.4. Effect of Tofacitinib and Anti-TNF on Thrombus Dynamics

To evaluate the effect of the treatments on hemostasis, blood coagulation was analyzed ex vivo for the different groups in the presence of tofacitinib, anti-TNF, or no drug using ROTEM. Extrinsic activation with tissue factor (EXTEM) and intrinsic activation with tissue factor (INTEM) assays were performed evaluating the hemostatic parameters MCF, CFT, and CT. No significant differences were observed in these parameters between the different experimental conditions in the three experimental groups (aUC, qUC, and HC) in both the EXTEM (Table 2) and INTEM (Table 3) assays.

## 3. Discussion

This is the first study evaluating the effect of tofacitinib on platelet function and coagulation in UC patients, in comparison with the anti-TNF agent infliximab. The ex vivo approach used in this study allows for better control of experimental conditions and minimizes inter-individual variability, thereby enhancing the reliability of our findings. Furthermore, evaluating patients in remission has allowed us to explore whether thrombotic risks in UC patients are a consequence of active inflammation or are inherently related to the disease or to treatment strategies. The goal was to pinpoint whether the prothrombotic susceptibility observed in UC, potentially due to treatment with tofacitinib, might necessitate the use of antiaggregant or anticoagulant therapy as part of patient management.

Our results demonstrate that both tofacitinib and anti-TNF increase the expression of platelet activation markers, such as CD63 and the PAC-1 binding antigen, in both aUC and qUC patients. This increase appears to result from the direct addition of these drugs to platelets in the ex vivo setting. However, these changes did not lead to measurable alterations in primary hemostatic functions, such as platelet aggregation. Notably, when TRAP, a specific agonist peptide for protease activated receptor (PAR)-1 and PAR-4, was added to platelet-rich plasma (PRP), differences in platelet activation between the groups were mitigated except for the patients with aUC. This finding suggests that prior drug exposure might modulate the response to TRAP stimulation through platelet desensitization or partial inhibition of specific signaling pathways.

Despite the increase in platelet activation observed with both drugs (tofacitinib and anti-TNF), no significant differences in aggregation were detected under the conditions tested. These results indicate that tofacitinib- and anti-TNF-induced increases in platelet activation are not sufficient to translate into changes in aggregation capacity, at least under the ex vivo conditions set in this study. It is possible that this is due to the fact that, despite the aggregometry and cytometry experiments being performed in PRP, the experimental conditions used in each were different. The unique effect of tofacitinib in the TRAP-induced dense granules release from platelets of aUC is not striking because the release of platelet granules is not uniform [23]. ROTEM analysis revealed no significant differences in clot formation parameters (in EXTEM and INTEM assays) between the treatment groups (tofacitinib, anti-TNF or no drug) in any of the patient groups (HC, qUC, or aUC). These results suggest that neither tofacitinib nor anti-TNF is able to substantially alter the dynamics of clot formation or the viscoelastic properties of the clot under the experimental conditions of these coagulation assays.

The clinical implications of our findings could be particularly relevant for treatment decision-making in UC patients receiving tofacitinib. Our study provides evidence that tofacitinib does not significantly alter primary hemostatic function or coagulation under ex vivo conditions, supporting its continued use without an immediate need for anticoagulant or antiplatelet co-therapy in this context.

The implications of these findings support the notion that the prothrombotic risks associated with UC are complex and may not be directly exacerbated by tofacitinib in the settings evaluated in this study. Moreover, they indicate that tofacitinib does not appear to significantly compromise primary hemostatic or coagulation processes in UC patients. Given the absence of studies directly addressing these aspects, our findings fill an important gap in the literature and offer a basis for further exploration. While the data suggest a lack of increase in thrombotic risks under tofacitinib treatment, this conclusion should be interpreted cautiously due to the limitations of our study.

We recognize that the ex vivo design of our study may not fully capture the complexity of in vivo physiological responses. The interactions between platelets, endothelial cells, and the immune system in a live organism could lead to different outcomes. Furthermore, the ex vivo nature of the study limits the assessment of potential confounding factors such as the influence of inflammatory cytokines or changes in the vascular environment, which could alter the results under in vivo conditions. Potential biases could also arise from the controlled nature of the ex vivo setting, as it does not account for the systemic effects of drugs or disease that might modulate platelet function and coagulation in a more dynamic, whole-body context.

The sample size of our study, with 30 patients, is relatively small and may limit the statistical power of our results, potentially reducing our ability to detect subtle differences between groups. To mitigate this limitation, we carefully controlled experimental conditions and employed paired analyses within the same individuals, minimizing inter-individual variability. Nevertheless, larger studies are needed to validate these findings and improve their generalizability.

Our results align with existing literature suggesting that tofacitinib does not increase thrombotic risk in UC patients [6,24], in contrast with some concerns raised in other immune-mediated diseases [8,14]. These discrepancies may be attributed to multiple factors. First, a key difference lies in the study population. While our study focuses on UC patients, previous reference studies analyze thromboembolic risk in rheumatoid patients. Second, our study employs an ex vivo approach, directly evaluating platelet function and coagulation through aggregometry, flow cytometry, and ROTEM analyses in samples from UC patients. In contrast, previous studies analyzed the incidence of thromboembolic events, without including direct assessment of platelet activation or coagulation. The differences observed between diseases such as rheumatoid arthritis and UC highlight the importance of disease-specific investigations and caution against broad extrapolation of data across distinct patient populations. These findings underscore that the thrombotic risk profile of therapies may not be universally applicable and emphasize the importance of tailored evaluations for each disease context.

## 4. Material and Methods

### 4.1. Study Design

**Figure 4:**
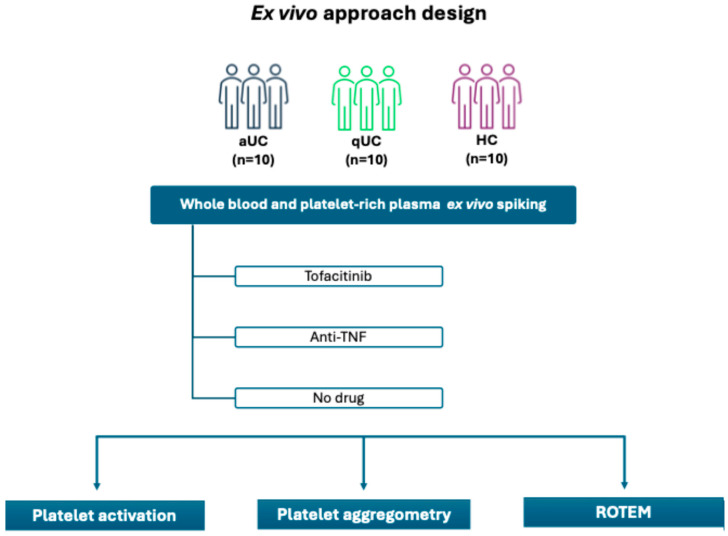
Study design. Tofacitinib or anti-TNF, in phosphate-buffered saline, was spiked on whole blood samples and platelet-rich plasma from the different study groups. Volumes spiked to whole blood samples and platelet-rich plasma were equal for all the treatments and experimental groups. HC: healthy control; aUC: active ulcerative colitis; qUC: quiescent ulcerative colitis; ROTEM: rotational tromboelastometry.

A total of 30 participants were included in the study: 10 aUC patients, 10 qUC patients, and 10 HC. The study was approved by the Ethics Committee of Hospital Universitario de La Princesa (GIS-2021-JAKihemo). All participating patients provided written informed consent, and all data were anonymized to ensure confidentiality. Figure 4 summarizes the study design.

The inclusion criteria were as follows: (1) UC patients: age 18 years or older, with diagnosis of UC according to the criteria of the European Crohn’s and Colitis Organisation (ECCO). Concomitant treatments were allowed if the treatment had remained stable for at least three months prior to the study. For active UC patients, endoscopic activity within one month after starting the treatment was required (Mayo endoscopic sub-score ≥ 2). (2) HC: Individuals without UC or any other inflammatory, allergic, malignant, or autoimmune disease.

The exclusion criteria were as follows: (1) UC patients: age under 18 years; presence of an immune-mediated disease, neoplasm, or active infection; pregnancy or breastfeeding; alcohol or drug abuse; ostomy; abdominal surgery in the last 6 months; colectomy; active infection by hepatitis B, C or human immunodeficiency virus (HIV); medical history of thromboembolic events; treatment with anticoagulants, antiplatelets, or other medications affecting blood coagulation; use of combined hormonal contraceptives or hormone replacement therapy; and hereditary coagulation disorders. (2) HC: Age under 18 years; presence of advanced chronic disease or any condition that prevents the monitoring of the protocol of this study; pregnancy or breastfeeding; active infection with hepatitis B, C, or HIV virus; alcohol or drug abuse; medical history of thromboembolic events; ongoing treatment with anticoagulants, antiplatelets, or other medications that alter blood coagulation; use of combined hormonal contraceptives or hormone replacement therapy; and hereditary coagulation disorders.

### 4.2. Blood Collection and Processing

Blood samples were collected from each participant in 6 sodium citrate tubes (3.2%, 454332 BD Vacutainer, Greiner Bio-One International GmbH, Madrid, Spain). Prior to functional assays, whole blood or PRP from each patient was incubated at 37 °C for 30 min in gentle shaking under three different experimental conditions: (1) tofacitinib (2.9 mg/mL), (2) anti-TNF (infliximab) (10 mg/mL), and without any drug [phosphate-buffered saline (PBS) solution].

### 4.3. Evaluation of Platelet Activation by Flow Cytometry

Whole blood was centrifuged at 152 g for 15 min at 25 °C with 9 accelerations and 3 decelerations to obtain PRP. PRP was diluted 1:5 with wash buffer (137 mM NaCl, 2.7 mM KCl, 1 mM NaH_2_PO_4_, 20 mM HEPES, 0.1% glucose, and 0.1% bovine serum album; pH 7.4). Subsequently, PRP was incubated in the presence of tofacitinib or anti-TNF. Following incubation, PE-anti CD41 (5112-PE100T, BioCytex, Marseille, France), FITC-anti CD62P mAb (a marker of the release from platelet’s alpha granules), FITC-anti CD63 mAb (a marker of the release from platelet’s dense granules), and FITC-anti PAC-1, an mAb that only recognizes the active conformation of the fibrinogen receptor (555523, 557288, 340507, respectively; Biosciences, NJ, USA), were added for 20 min at room temperature. The samples were then diluted in PBS for flow cytometry (BD Canto II, BD Biosciences). Data were analyzed using FlowJo software 10.8 (Ashland, OR, USA). Platelet activation was assessed at baseline and after stimulation with TRAP (Roche Diagnostics, Rotkreuz, Switzerland).

### 4.4. Platelet Aggregation

Platelet aggregation was measured in PRP using a lumiaggregometer (Helena Biosciences Europe, Beaumont, TX, USA). PRP was prepared as described above, and platelet-poor plasma (PPP) was obtained by centrifugation of whole blood at 3250 rpm for 20 min at 25 °C. PPP served as the reference for equipment calibration, and PRP was adjusted to 200–300 × 10^3^ platelet/µL with PPP. PRP was incubated in the presence of tofacitinib, anti-TNF, or no drug (control group). Aggregation was induced by adding ADP at concentrations of 2.5 µM and 5 µM and measured for 10 min by light transmission aggregometry. Results were expressed as percentage of maximal aggregation.

### 4.5. Whole Blood Coagulation

Whole blood coagulation was assessed using rotational thromboelastometry ROTEM^®^ (TEM Innovations GmbH, Munich, Germany) according to the manufacturer’s instructions. Blood was collected in 3 sodium citrate tubes. One tube was incubated with tofacitinib, one with anti-TNF, and one with no drug. The assays used were EXTEM and INTEM. Maximum clot firmness (MCF), clot formation time (CFT), and clotting time (CT) were measured after ex vivo supplementation with tofacitinib or anti-TNF and without any drug (PBS buffer).

### 4.6. Sample Size

The sample size for the ex vivo study was 10 subjects for each experimental group (aUC, qUC, and HC). Based on previous studies, a sample size of 8 patients was considered capable of providing 80% power to detect a 2 mm difference in MCF, with a significance level of 0.05 and a standard deviation of 2 mm. An increase of 2 mm corresponded to approximately a 20% increase in MCF [25,26,27]. Therefore, for this pilot study involving comparisons between groups, a sample size of 10 subjects per group was deemed sufficient to detect changes in hemostasis. Furthermore, previous studies evaluating coagulation alterations using ROTEM parameters, as well as platelet function, included a similar sample size [25,28,29,30].

### 4.7. Statistical Analysis

Descriptive statistics were employed to report characteristics of the study population including age, sex, and smoking status (smoker or non-smoker). These data were presented as means ± standard deviations (SD) for continuous variables and as percentages for categorical variables.

The data were analyzed using Graph Pad Prism software (version 9.5.1; Graph pad Software Inc., San, CA, USA). Comparisons of ROTEM, platelet aggregation, and platelet activation data between the three groups (aUC, qUC, and HC) under three experimental conditions (ex vivo incubation with tofacitinib, anti-TNF, or no drug) were performed using two-way ANOVA. *p*-values < 0.05 were considered statistically significant.

## 5. Conclusions

This study provides valuable insights into the effects of tofacitinib and infliximab on platelet activation and coagulation in UC patients. Although both tofacitinib and anti-TNF increased platelet activation markers, they did not significantly affect platelet aggregation or coagulation under our ex vivo experimental conditions. These findings suggest that, at least in the settings tested, tofacitinib and anti-TNF therapies do not significantly alter hemostatic function. Further research, including larger studies and in vivo models, is needed to better understand the long-term thrombotic risks and refine treatment strategies for UC patients.

## Figures and Tables

**Table 1:** Baseline clinical characteristics of the study population.

Scheme	HC	aUC	qUC	*p*-Value
Age (years), mean (SD)	55 (22)	41 (17)	59 (11)	0.06
Female, n (%)	6 (60)	5 (50)	6 (60)	0.99
Smokers, n (%)	1 (10)	2 (20)	1 (10)	0.99
BMI, mean (SD)	24 (4)	24 (6)	25 (3)	0.71

Differences were analyzed using the one-way ANOVA test. HC: healthy control; aUC: active ulcerative colitis; qUC: quiescent ulcerative colitis; BMI: body mass index; SD: standard deviation.

**Table 2:** Effect of tofacitinib and anti-TNF on blood coagulation in assays with extrinsic activation with tissue factor (EXTEM).

Study Groups	EXTEM Assay								
	CT (Seconds)			CFT (Seconds)			MCF (mm)		
	aUC	qUC	HC	aUC	qUC	HC	aUC	qUC	HC
Tofacitinib, mean (SD)	63 (5)	63 (8)	62 (9)	103 (21)	128 (34)	97 (39)	56 (4)	54 (5)	57 (7)
Anti-TNF, mean (SD)	62 (5)	59 (5)	63 (5)	83 (15)	95 (26) *	96 (34)	59 (3)	58 (4)	57 (7)
No drug, mean (SD)	63 (8)	63 (8)	65 (16)	127 (36)	157 (73)	109 (41)	54 (4)	52 (5)	56 (7)

Table 2 shows rotational tromboelastometry (ROTEM) parameters from EXTEM assays with blood samples from different participant groups (aUC, qUC, and HC) in the presence of tofacitinib, anti-TNF, or without drug. Differences were analyzed using the two-way ANOVA test. * Statistically significant differences between anti-TNF and no drug (*p*-value = 0.0292). CT: clotting time; CFT: clot formation time; MCF: maximum clot firmness; HC: healthy control; aUC: active ulcerative colitis; qUC: quiescent ulcerative colitis; SD: standard deviation.

**Table 3:** Effect of tofacitinib and anti-TNF on blood coagulation in assays with intrinsic activation (INTEM).

Study Groups	INTEM Assay								
	CT (Seconds)			CFT (Seconds)			MCF (mm)		
	aUC	qUC	HC	aUC	qUC	HC	aUC	qUC	HC
Tofacitinib, mean (SD)	149 (14)	141 (17)	151 (15)	95 (20)	109 (30)	91 (36)	55 (3)	38 (6)	55 (7)
Anti-TNF, mean (SD)	168 (20)	163 (17)	162 (9)	79 (11)	86 (20)	88 (26)	58 (6)	57 (5)	55 (6)
No drug, mean (SD)	148 (23)	146 (18)	151 (16)	105 (25)	130 (47)	94 (31)	55 (3)	52 (4)	54 (7)

Table 3 shows rotational tromboelastometry (ROTEM) parameters from INTEM assays with blood samples from different participant groups (aUC, qUC, and HC) in the presence of tofacitinib, anti-TNF, or without drug. Differences were analyzed using the two-way ANOVA test. CT: clotting time; CFT: clot formation time; MCF: maximum clot firmness; HC: healthy control; aUC: active ulcerative colitis; qUC: quiescent ulcerative colitis; SD: standard deviation.

## Data Availability

All data generated during this study are included in the article.

## Abbreviations

ADP	adenosine diphosphate
anti-TNF	anti-tumor necrosis factor
aUC	active ulcerative colitis
HC	healthy control
HIV	human immunodeficiency virus
JAK	Janus kinase
PPP	Platelet-poor plasma
PRP	Platelet-rich plasma
qUC	quiescent ulcerative colitis
ROTEM	rotational tromboelastometry
STAT	signal transducer and activator of transcription
TRAP	thrombin receptor activating peptide
UC	ulcerative colitis
